# Poly(butylene succinate-*co*-butylene acetylenedicarboxylate): Copolyester with Novel Nucleation Behavior

**DOI:** 10.3390/polym13030365

**Published:** 2021-01-24

**Authors:** Yi Li, Guoyong Huang, Cong Chen, Xue-Wei Wei, Xi Dong, Wei Zhao, Hai-Mu Ye

**Affiliations:** 1 Department of Materials Science and Engineering, China University of Petroleum, Beijing 102249, China; liyi_cup@163.com (Y.L.); huanggy@cup.edu.cn (G.H.); 18401682407@163.com (C.C.); wxw8383@163.com (X.-W.W.); dx2020669@163.com (X.D.); 2 Beijing Key Laboratory of Failure, Corrosion and Protection of Oil/Gas Facilities, China University of Petroleum, Beijing 102249, China; 3 SCNU-TUE Joint Lab of Device Integrated Responsive Materials (DIRM), National Center for International Research on Green Optoelectronics, South China Normal University, Guangzhou Higher Education Mega Center, Guangzhou 510006, China

**Keywords:** poly(butylene succinate), copolymer, crystallization behavior, nucleation mechanism

## Abstract

Big spherulite structure and high crystallinity are the two main drawbacks of poly(butylene succinate) (PBS) and hinder its application. In this work, a new type of copolyester poly(butylene succinate-*co*-butylene acetylenedicarboxylate) (PBSAD) is synthesized. With the incorporation of acetylenedicarboxylate (AD) units into PBS chains, the crystallization temperature and crystallinity are depressed by excluding AD units to the amorphous region. In contrast, the primary nucleation capability is significantly strengthened, without changing the crystal modification or crystallization kinetics, leading to the recovery of total crystallization rate of PBSAD under the same supercooling condition. The existence of specific interaction among AD units is found to be crucial. Although it is too weak to contribute to the melt memory effect at elevated temperature, the interaction continuously strengthens as the temperature falls down, and the heterogeneous aggregation of AD units keeps growing. When the aggregating process reaches a certain extent, it will induce the formation of a significant amount of crystal nuclei. The unveiled nucleation mechanism helps to design PBS copolymer with good performance.

## 1. Introduction

Poly(butylene succinate) (PBS) as one of the promising biodegradable polyesters, can exhibit comparable mechanical properties with traditional polyolefins [1,2], showing great potential in diversified applications of packing materials, clothing, biomedical engineering, etc., [3,4]. At the same time, PBS suffers some severe drawbacks, including large spherulite structure, high crystallinity, and serious post-crystallization, which make it brittle and weak in impact resistance [5].

Nucleating agent is usually blended into PBS matrix to improve the heterogeneous nucleation that contributes to the reduction of spherulite size. Various nucleating agents including organic molecules [6,7], inorganic particles [8,9], nanofillers [10,11] have been used to effectively decrease the spherulite size and improve the mechanical performance. Nevertheless, the problem of high crystallinity or post-crystallization behavior cannot be avoided [12,13], which makes the PBS composites still brittle in character. On the other hand, copolymerization is an effective approach to resolve the high crystallinity and post-crystallization problems of PBS. When comonomers, like adipic acid [14], terephthalic acid [15], 1,3-propane diol [16], and ethane diol [17], are copolymerized with PBS, the crystallinity of copolymer can be notably decreased depending on the comonomer content. However, in many reports, the spherulite sizes of PBS copolymers are commonly larger than or close to that of homo-PBS [18,19,20,21]. Therefore, the simultaneous introduction of nucleating agent and comonomer unit are required to grant the PBS-based materials with good performance [22,23].

When special comonomer units are incorporated into the homo-polymer chains, specific pre-ordered conformation or heterogeneous aggregation will appear in the melt, such as the incorporation of butylene fumarate (with fixed *trans* conformation) into PBS [24] and diethanolamine hydrochloride (with strong ionic interaction) into poly(ethylene succinate) (PES) [25]. The capability to nucleate was found to be promoted with or without decreasing the crystallinity. The detailed mechanism still needs to be further clarified. For example, is it through strengthening the melt memory effect that the nucleation is promoted upon introducing of comonomer units?

In this study comonomers dimethyl acetylenedicarboxylate (DMAD) were copolymerized with PBS to obtain a new type of copolyester, poly(butylene succinate-*co*-butylene acetylenedicarboxylate) (PBSAD), which contains special and rigid C≡C structure in the main chains. The crystallization behavior of PBSAD was systematically studied and compared with PBS. It was interesting to find that the primary nucleation capability was significantly enhanced while the crystallinity was simultaneously lowered in PBSAD. Based on these results, the nucleating mechanism was unveiled in detail.

## 2. Materials and Methods

### 2.1. Materials

Succinic acid (SA, AR grade), dimethyl acetylenedicarboxylate (DMAD, AR grade), 1,4-butane diol (BDO, AR grade), and the catalyst tetrabutyl titanate (TBT, AR grade) were all purchased from Shanghai Aladdin Industrial Inc. (Shanghai, China). All reagents were used without further purification.

### 2.2. Synthesis of PBS and PBSAD

PBS and PBSAD were synthesized through a two-stage reaction. For the first stage, the desired amounts of SA, DMAD, BDO were charged into a three-necked flask under high purity nitrogen atmosphere, and stirred with a Teflon-coated magneton. The molar ratio of BDO:(SA + DMAD) was set constantly at 1.3:1, while the ratios of SA:DMAD were chosen as 1:0, 0.95:0.05, 0.9:0.1, and 0.8:0.2, respectively. The system was scheduled to react with a step heating process from 125 to 200 °C with a temperature interval of 25 °C and duration of 60 min at each temperature step. For the second stage, TBT with 1 wt % of all reactants was added and the temperature was heated to 225 °C for further polycondensation reaction under high vacuum. The magneton kept rotating and slowed down with the increase of viscosity of polyester melt. After 60 min, the magneton reached a very slow rotating state, then the reaction was stopped. The resulting product was completely dissolved into chloroform, and centrifuged at a rate of 10,000 rpm for 15 min to separate the insoluble impurity. After that, the resulting clear solution was precipitated with excess amount of cold methanol, followed by a filtration operation to obtain the final polyester, which was further dried in a vacuum oven at 60 °C for 1 day.

### 2.3. Characterization

The chain compositions of PBS and PBSAD were measured on a JNM-ECA600 nuclear magnetic resonance spectroscopy (NMR, Tokyo, Japan) with deuterated chloroform (CDCl_3_) as the solvent and tetramethylsilane (TMS) as the standard. The weight-average molecular weights (*M*_w_) and dispersity indices (*Ð*) of the synthesized polyesters were measured by a Waters 515 HPLC gel permeation chromatography (GPC, Milford, US), and HPLC grade chloroform was used as the eluent. The Raman spectra of polyesters were obtained on a Horiba JY HR-800 Raman microscope system (Paris, France) and collected by a charge-coupled device (CCD) detector (Paris, France). The laser wavelength was selected as 532 nm, and the employed power was adjusted not to damage the testing samples. The Fourier infrared (FTIR) spectra of polyesters were collected on a Bruker Hyperion spectrometer (Karlsruhe, Germany) installed with an accessory of Linkam THMS600 hot-stage (Surrey, UK). All samples for FTIR measurement were first prepared as a film of ~10 μm in thickness on a CaF_2_ wafer. Differential scanning calorimetric (DSC) test was performed on a Netzsch-204F1 (Berlin, Germany) to unveil the non-isothermal, isothermal crystallization behavior and the memory effect in the melt of polyesters. The temperatures for isothermal crystallization were selected by referring to the onset crystallization temperatures of non-isothermal crystallization curves and using an increasing interval of 2 °C. The crystal structures of polyesters were investigated on a Bruker D8 Focus X-ray diffractometer (Karlsruhe, Germany) operated at 40 kV and 200 mA with a Cu *K*α radiation. The scan was carried at a range of 15°–40° with a scanning rate of 2°/min, and the step interval was set as 0.01°. Spherulite morphology of polyesters was observed with a Leica DM-2500P polarized optical microscope (POM, Wetzlar, Germany) equipped with a Linkam THMS600 hot-stage (Surrey, UK). Typical morphology was captured for further discussion.

## 3. Results

### 3.1. Chain Structure

**Figure 1:**
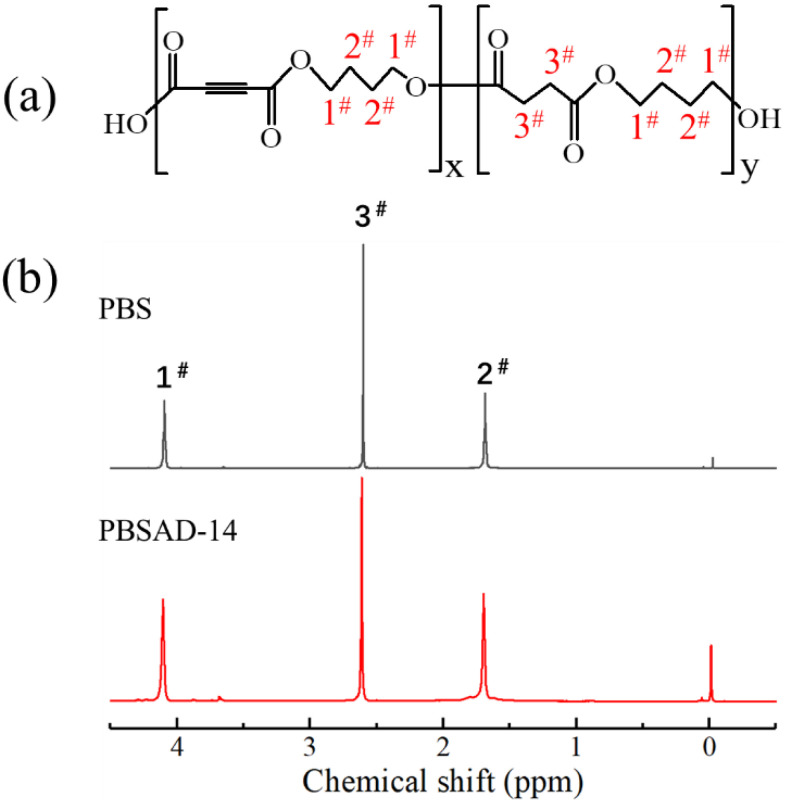
(**a**) Chemical structure of poly(butylene succinate-*co*-butylene acetylenedicarboxylate) (PBSAD) and (**b**) the ^1^H NMR spectra of poly(butylene succinate) (PBS) and PBSAD-14.

Figure 1 and S1(in the Supplementary Materials) shows the ^1^H NMR spectra of PBS and PBSAD. They display the same main peaks of protons with possibly slight shift, since no H atom exists in the alkyne (C≡C) group. The peak 1# and 2#, locating at 4.09 and 1.68 ppm respectively, correspond to the H atoms in the side and central CH_2_ groups. The peak 3# at 2.60 ppm can be assigned to the H atoms in succinic units. It is clear that the incorporation of acetylenedicarboxylic (AD) units into PBS chains would decrease the intensity of peak 3# when compared with peak 1# or peak 2#. Therefore, it is possible to calculate the molar content of acetylenedicarboxylic unit (*P*_AD_) in the copolyester chains by using Equation (1).

(1) $$P_{\mathrm{AD}}=\frac{I_{\mathrm{peak}\;1\#}-I_{\mathrm{peak}\;3\#}}{I_{\mathrm{peak}\;1\#}}\times 100\;\mathrm{mol}\%$$

where “*I*” represents the intensity of the peak in NMR spectrum.

The calculated compositions are tabulated in Table 1, and the samples are named by referring to the comonomer contents. In addition, the molecular weights and dispersity indices are shown in Table 1 too.

**Figure 2:**
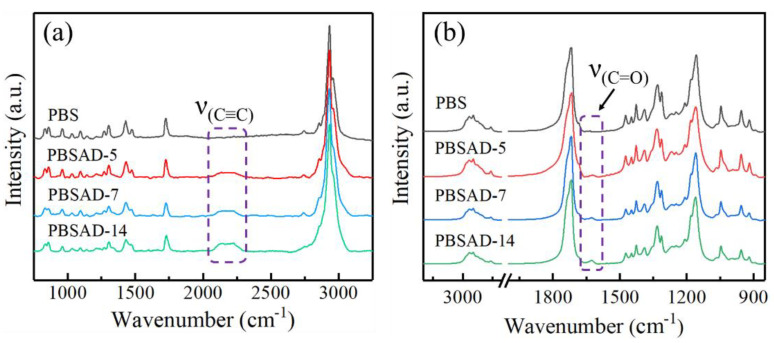
(**a**) Raman spectra and (**b**) FTIR spectra of PBS and PBSAD.

The Raman and FTIR spectra were also employed to confirm the incorporation of AD units in the polyester chains. The Raman spectroscopy is sensitive to the vibration of nonpolar alkyne C≡C structure. As shown in Figure 2a, after copolymerization of DMAD, the characteristic absorption bands due to the stretching vibration of the triple bonds at wavenumbers of 2226–2140 cm^–1^ emerge [26,27,28] and gradually strengthen with respect to AD content. In the FTIR spectra (Figure 2b), an additional absorption peak at ~1623 cm^-1^ appears and its intensity increases with AD content. According to literature [29,30], such a peak could be attributed to the stretching vibration of carbonyl (C=O) groups which are dramatically affected by the adjacent electron-donating groups like C≡C. Based on the above results, it is clear that the copolyesters of PBSAD were successfully obtained. 

### 3.2. Non-Isothermal and Isothermal Crystallization

**Figure 3:**
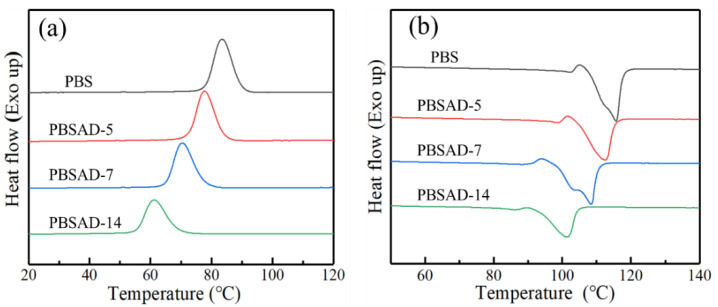
DSC thermograms of polyesters (**a**) being cooled from melt and (**b**) the subsequent heating process at a rate of 10 °C/min.

To investigate the effect of AD units on the crystallization behavior of PBS, non-isothermal crystallization was first performed at a rate of 10 °C/min. In Figure 3a, the crystallization peak (*T*_c_) of PBS at 83.4 °C shifts to lower temperatures with increasing AD content, and reaches 61.1 °C for PBSAD-14 (Table 1). Meanwhile, the area of crystallization peak becomes smaller, that is, the exothermal enthalpy (Δ*H*_c_) decreases. It can be concluded that the AD units imped the crystallization ability of PBS and should be preferably excluded to the amorphous region. During the subsequent heating process, both the melting point (*T*_m_) and the melting enthalpy (Δ*H*_m_) of polyester decline with increasing incorporation content of AD. The *T*_m_ and Δ*H*_m_ of PBSAD-14 are lowered down to 101.1 °C and 50.5 J/g, which are close to the values of other random copolymers of PBS, such as poly(butylene succinate-*co*-butylene adipate) (PBSA) [31], poly(butylene succinate-*co*-propylene succinate) (PBSP) [32], and poly(butylene succinate*co*butylene terephthalate) (PBST) [33]. The similar extent of decline for the thermal properties further confirms that the copolymerized AD units are excluded out of the crystalline region.

**Figure 4:**
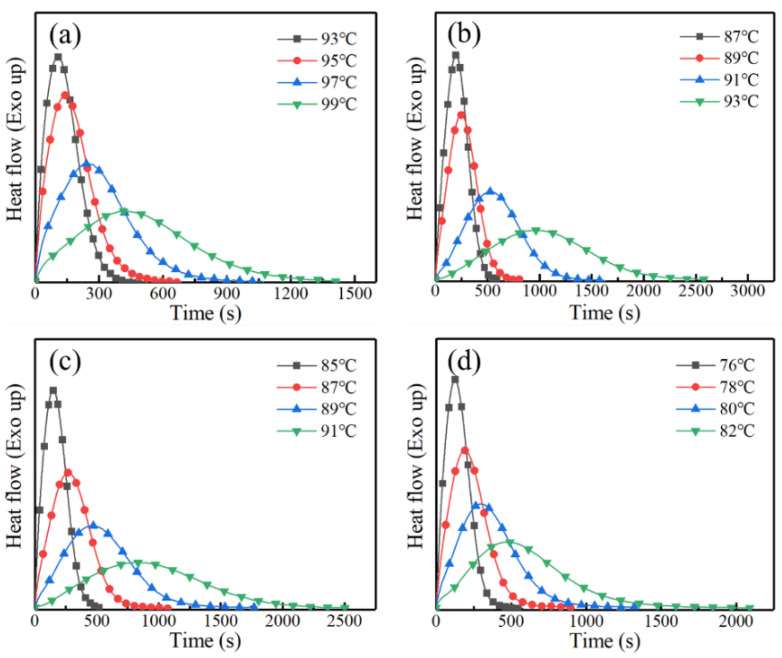
The time-dependent exothermal curves of polyesters isothermally crystallized at specified temperatures. (**a**) PBS, (**b**) PBSAD-5, (**c**) PBSAD-7, and (**d**) PBSAD-14.

Isothermal crystallization measurement was also carried out to demonstrate the effect of AD unit on the crystallization ability and kinetics of PBS. The time-dependent exothermal curves of four polyesters isothermally crystallized at suitable temperatures are illustrated in Figure 4. As the crystallization temperature increases, the exothermal process becomes longer. Through integrating and normalizing procedures, these curves are transformed into a new set of S-shape plots of relative crystallinity (*X*_t_) over time (*t*), which are used for further calculation of the crystallization kinetics parameters Figure S2 (in the Supplementary Materials).

Avrami method (Equation (2)) was employed to deduce the crystallization kinetics parameters of PBS and PBSAD based on the relationship between *X*_t_ and *t* [34,35]:

(2) $$1-X_{t}=\exp\left(-kt^{n}\right)$$

where “*n*” and “*k*” correspond to the Avrami index and the crystallization rate constant. The fitting operation is presented in Figure S3 (in the Supplementary Materials) and the resulted data are summarized in Table 2.

The Avrami indices of PBSAD remain almost the same as PBS at around 2, plausibly implying that the all four polyesters studied here adopt the same crystallization behavior of two-dimensional spherulitic growth with athermal nucleation mechanism. Incorporation of AD units into PBS chains does not change the crystallization mechanism. The value of *k* decreases with respect to the selected crystallization temperatures, indicating nucleation is the more dominant factor than the diffusion of chains.

**Figure 5:**
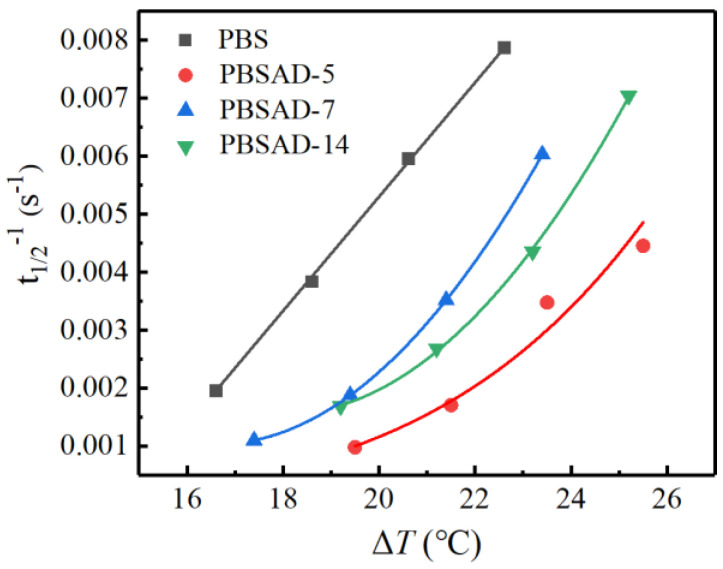
The plots of total crystallization rates vs. supercooling degrees of PBS and PBSAD.

The total crystallization rates (*G*_total_) of polyesters were evaluated based on the half crystallization times (*t*_1/2_). The reciprocals of the half crystallization times (*t*_1/2_^–1^ = *G*_total_) were calculated and plotted with the corresponding supercooling degrees (Δ*T* = *T*_m_ – *T*_c_) in Figure 5.

When 5 mol % AD units are incorporated into the PBS chains, the crystallization rate declines under the same Δ*T* conditions. However, the crystallization rate recovers and shows less decline when more AD units are incorporated, i.e., 7 mol %, which is abnormal and different from other copolymers of PBS [19,36] and other polymers [37,38]. Since AD units are not favored in the crystalline structure and preferably excluded into the amorphous region, the spherulite growth is supposed to be suppressed. The unexpected recovery of total crystallization rate in PBSAD-7 presumably stems from the enhancement of primary nucleation capability. Further increase of AD content to 14 mol% leads to slight decrease of crystallization rate, which is attributed to the balancing result of primary nucleation and spherulite growth. Overall, the crystallization rate of PBSAD-14 is still higher than PBSAD-5 under the same Δ*T* conditions, further implying the AD unit will lead to enhanced primary nucleation.

### 3.3. Crystal Morphology and Structure

**Figure 6:**
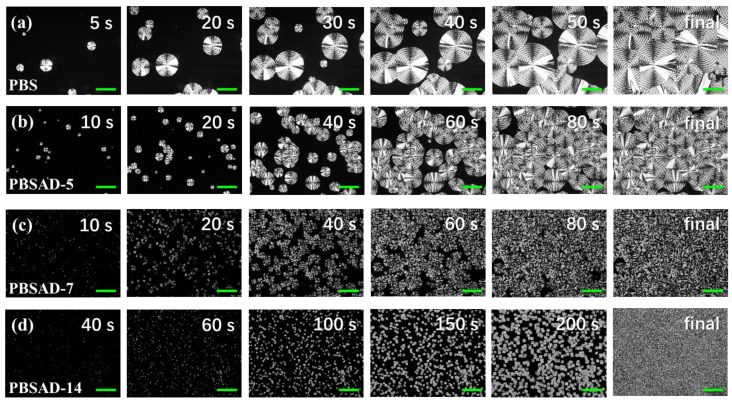
POM images of polyester spherulites grown at 80 °C. (**a**) PBS, (**b**) PBSAD-5, (**c**) PBSAD-7, and (**d**) PBSAD-14. All scale bars are 100 μm in length. The melt was quickly transferred from melt to 80 °C within 1 s.

To clarify the working mechanism of AD unit on the crystallization of PBS, all four polyesters were isothermally crystallized at 80 °C after melting at 150 °C for 3 min. The real-time POM observation of crystallization processes was carried out, and the representative images of morphology were captured and are shown in Figure 6. PBS displays scattered primary nuclei after the melt reached 80 °C that further grow in typical spherulite morphology showing Maltese cross pattern with banded and non-banded region in one spherulite [4,39]. The nucleation density increases in PBSAD-5, while the spherulite growth rate decreases compared with PBS, 1.67 μm/s versus 3.61 μm/s. Further increase of AD units in the copolyester chains make the nucleation density increase remarkably, i.e., in PBSAD-7 and PBSAD-14. As a result, the recovery of total crystallization rate after incorporating more than 5 mol% of AD units, as detected by DSC under the same Δ*T* conditions, could be firmly attributed to the notable enhancement of primary nucleation.

From the images of polyesters completely crystallized at 80 °C, the final nucleation densities are estimated, and thus the average equivalent diameters (*d*) are obtained (Table 3). The value of *d* decreases from ~151 μm to ~89 μm when 5 mol% AD units are copolymerized into PBS chains, and sharply drops to ~34 μm and ~21 μm in PBSAD-7 and PBSAD-14, respectively. The diameters are even much lower than the corresponding poly(butylene succinate-*co*-butylene fumarate) (PBSF) (Figure S4 (in the Supplementary Materials)). The fumarate units in PBSF keep the same conformation in melt as in the crystalline region, which is very beneficial for reducing entropic barrier hence promoting primary nucleation [24]. It is worth emphasizing the remarkable promotion effect of AD units on the nucleation of PBS crystals.

**Figure 7:**
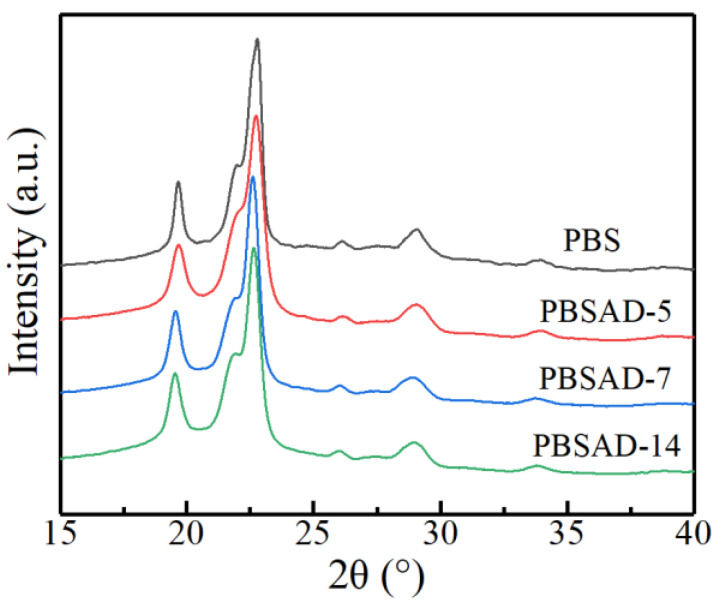
Wide angle X-ray diffractograms of synthesized PBS and PBSAD.

The wide angle X-ray diffractograms of melt-crystallized PBS and PBSAD are depicted in Figure 7. All PBSADs exhibit the same diffraction peaks at 2θ = 19.6°, 22.0°, 22.8°, 26.1°, 29.1°, and 33.9° as PBS, indicating they all adopt the α crystal modification of PBS [6,40]. The AD units do not change the crystal structure of polyester, and the enhanced nucleation capability is not originated from different crystal modifications.

### 3.4. Evaluation of Melt Memory Effect

**Figure 8:**
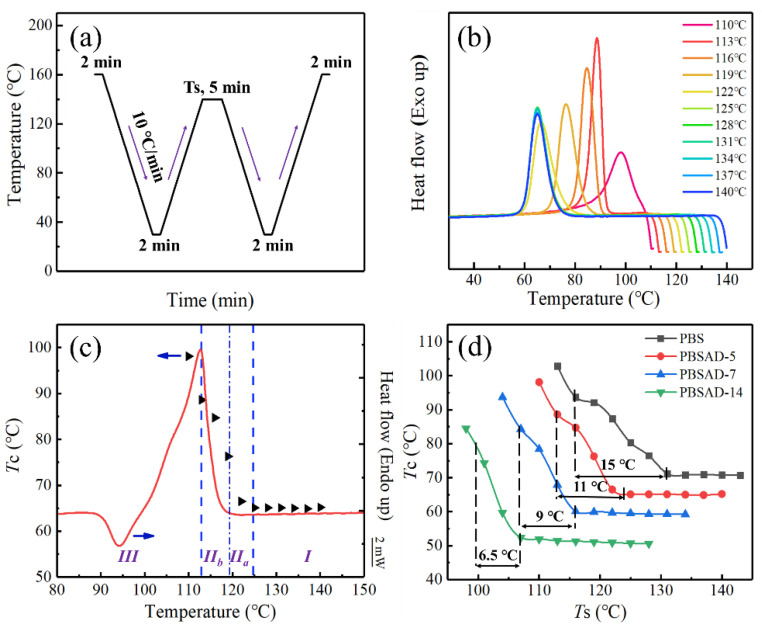
(**a**) Schematic representation of melt memory effect measurement procedure, and (**b**) the cooling DSC curves of PBSAD-5 after being melted at various *T*_s_ for 5 min. (**c**) A typical plot of the domains for PBSAD-5 (the red, solid line is the melting DSC curve, the solid triangles represent the crystallization temperatures obtained after melting at different *T*_s_). (**d**) The crystallization temperature trends of PBS and PBSAD after melting at different *T*_s_ (the temperature intervals marked by the dotted lines are the melt memory range of samples).

Since the crystallization kinetics and crystal modification of PBSAD are consistent with PBS and the AD units are excluded from the crystalline region, mechanism for the significantly enhanced nucleation capability of PBSAD still remains unclear. According to the work of Zhou’s and Müller’s groups [41,42], PBS could exhibit obvious memory effect that result in a significant increase of nucleation density. The memory effect of chain conformation in melt might be the key factor that promotes the formation of primary nuclei. Hence a specially designed DSC measurement procedure (Figure 8a) was carried out for PBS and PBSAD to evaluate the melt memory effect.

By melting samples at various setting temperatures (*T*_s_), a series of subsequent cooling DSC curves can be shaped. As shown in Figure 8b, when PBSAD-5 is melted at 110 °C, it displays a broad and high *T*_c_ at 98 °C. The crystallization peak becomes narrow and shifts to lower temperature with increasing *T*_s_, then remains almost unchanged after the value of *T*_s_ exceeds 125 °C, implying the memory effect is completely eliminated. The cooling DSC curves of other three polyesters are presented in Figure S5 (in the Supplementary Materials).

To evaluate the melt memory effect of PBS and PBSAD, the method shown in Figure 8c is used to partition the nucleation phenomenon of the samples [42]. For PBSAD-5, there are four specific domains. Domain *I* is the heterogeneous nucleation domain, where the melt is isotropic. The nucleation density during crystallization is low and constant. The crystallization behavior is dominated by common heterogeneous nucleation, and there is no melt memory effect or self-nucleation phenomenon. Domain *II*_a_ is the self-nucleation domain, located above the melting end temperature. At these temperatures the crystal has been completely melted, but there are still some partially ordered segments. The crystallization process is dominated by the homogeneous nucleation produced by the melt memory effect, and the nucleation density increases. Domain *II*_b_ is the self-seeding domain, located between the melting point and the melting end temperature. In these cases, a small number of crystals have not completely melted, which act as nuclei during the cooling process, leading to a sharp increase in the nucleation density. Domain *III* is the self-nucleation and annealing domain, where only a small part of the crystals is melted. The remaining crystals will continue to thicken or undergo an annealing process at the set melting temperature.

The melt memory effect can be clearly understood based on the analysis, and it only exists in the domain *II*, namely the *II*_a_ and *II*_b_ domains. When the selected *T*_s_ is in domain *II*, self-nucleating will occur because of insufficient melting of crystals. The number of nucleation will increase significantly during the subsequent cooling process, that is, the melt memory effect will appear. The relationships between *T*_c_ and *T*_s_ are plotted in Figure 8d, and the exact temperature ranges are listed in Table 4. With the increase of AD units in chains, the range of domain *II* gradually narrows down from 15.0 °C for PBS to 6.5 °C for PBSAD-14, indicating the gradual weakening of the melt memory effect.

After dividing the domain *II* into domains *II*_a_ and *II*_b_, it is interesting to notice that the domain *II*_b_ remains unchanged while the domain *II*_a_ changes notably (Table 4). The result suggests the self-nucleation domain narrows with increasing AD content, implying the rigid C≡C structure of AD unit does not promote the formation of partially ordered segments in the melt. Overall, since the DSC and POM investigations are operated after the samples melt at 140 °C or even higher temperatures, which fall in the domain *I*, the melt memory effect is not responsible for the remarkable increase of nucleation density in PBSAD.

### 3.5. In Situ FTIR Investigation

**Figure 9:**
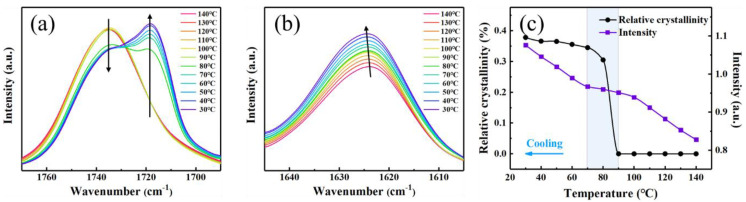
In situ FTIR spectra of PBSAD-14 during melt cooling process at (**a**) wavenumbers from 1780 cm^−1^ to 1690 cm^−1^, and (**b**) from 1645 cm^−1^ to 1605 cm^−1^. (**c**) The dependences of relative crystallinity of PBSAD-14 and absorption intensity of C=O stretching vibration in AD units are plotted as function of temperature during melt-cooling.

Since the difference between PBS and PBSAD is mainly due to the AD unit, and both the crystalline structure and AD unit can be detected using FTIR spectrum (Figure 2b), in situ FTIR was used to unveil the possible mechanism of enhanced nucleation capability of PBSAD. During the melt-cooling process of PBSAD-14, the intensity of C=O absorption band of succinate (SU) units at 1735 cm^−1^ keeps unchanged, while that of AD units at 1623 cm^−1^ grow up with decreasing temperature (Figure 9a,b). These results indicate the existence of weak interaction among AD units, similar to the observation of hydrogen-bonding interaction in polymer melt [43,44]. The slight shift of band 1623 cm^−1^ also confirms the existence of weak interaction [45]. The C≡C groups in AD units plausibly prefer to stack together or aggregate, which could reduce the electron-donating effect and induce the blue-shift of the specific C=O absorption band at 1623 cm^−1^. The intensity of this band at 1623 cm^−1^ is plotted against temperature to clearly visualize the rate of change (Figure 9c). When the melt is cooled from 90 to 70 °C, crystals form as the crystalline band at 1718 cm^−1^ appears and the amorphous band at 1735 cm^−1^ weakens. Meanwhile, the rate of intensity changes for band 1623 cm^−1^ slows down, which might be due to the restriction exerted on the aggregation of AD units due to the crystallization process. Polymer chains in melt are expected to be dragged during crystallization [46]. When the sample is further cooled after crystallization, the rate of change recovers to the same level as before crystallization. Such a recovery phenomenon confirms that the AD units are (all) in amorphous region after the crystallization.

**Figure 10:**
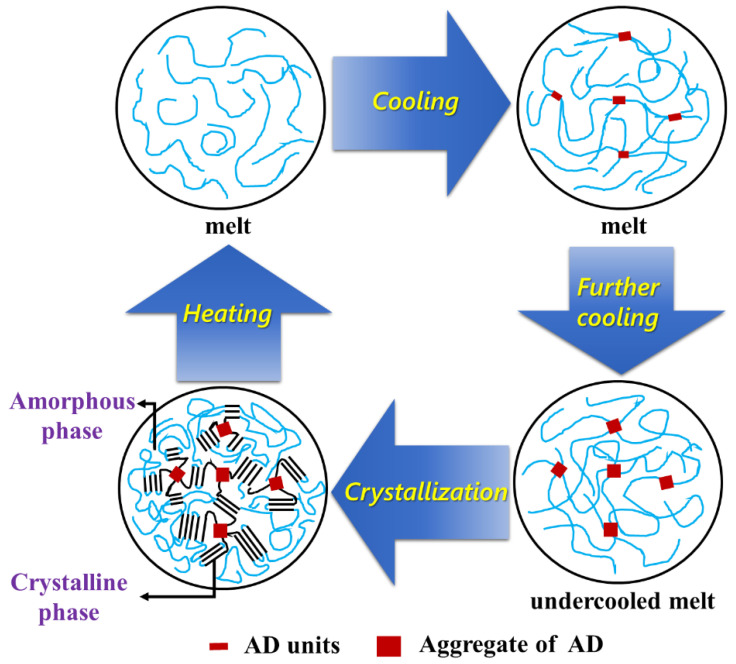
Schematic representation for the crystallization process of PBSAD.

Consequently, a nucleating mechanism of AD units in PBSAD could be proposed based on the study of melt memory effect and FTIR results, as sketched in Figure 10. After the AD units are incorporated into the PBS chains, in melt they tend to aggregate because of specific interaction between C≡C groups. Such interaction is weak at temperatures above melting point, which does not contribute to any melt memory effect. Instead, the melt memory effect is impaired possibly due to the dilution of AD units on the PBS chains. When the temperature is lowered, the interaction gradually strengthens and the aggregation of AD unit becomes stable and continues to grow. The heterogeneous aggregation of AD units, after reaching a certain extent, will induce a large number of primary nuclei to grow in spherulites. The higher content of AD unit is incorporated, the more aggregation would form, therefore the higher nucleation density is resulted in, which coincides with the DSC and POM observations.

## 4. Conclusions

Here, new copolyesters of PBSAD were synthesized by incorporating AD units into PBS chains, and the crystallization behavior was systematically studied. Incorporation of AD unit will lower the crystallization temperature during non-isothermal process and decrease the crystallinity without altering the mechanism of crystallization kinetics. However, the AD units, containing C≡C groups, could significantly enhance the primary nucleation, even at low copolymerization content. The nucleation capability continuously improves with respect to the content of AD units, which results in the recovery of total crystallization rate of PBSAD under the same supercooling condition, i.e., PBSAD-7 and PBSAD-14 display higher crystallization rate than PBSAD-5. Based on the investigation of melt memory effect and in situ FTIR spectra, the mechanism for the promoted nucleation capability due to AD units can be reasonably speculated. Specific interaction among AD units exists, although it is too weak to contribute to the melt memory effect at elevated temperature. The interaction continuously strengthens as the temperature decreases, and the heterogeneous aggregation of AD units gradually grows. When the aggregating process reaches a certain extent, it will induce the formation of a significant amount of crystal nuclei. By incorporating the AD units, improvement of nucleation and reduction of crystallinity can be simultaneously achieved, which could help develop PBS materials with good performance.

## Figures and Tables

**Table 1:** Chain compositions, weight–average molecular weights (*M*_w_), dispersity indices (*Ð*), crystallization temperatures (*T*_c_) and enthalpy (Δ*H*_c_), melting temperatures (*T*_m_) and enthalpy (Δ*H*_m_) of synthesized polyesters.

Sample	*P*_AD_	*M*_w_ (g/mol)	*Ð*	*T*_c_ (°C)	Δ*H*_c_ (J/g)	*T*_m_ (°C)	Δ*H*_m_ (J/g)
PBS	0	4.45 × 10^4^	2.48	83.4	68.4	114.6	70.2
PBSAD-5	5 mol%	2.07 × 10^4^	1.73	77.7	63.0	112.5	63.8
PBSAD-7	7 mol%	1.78 × 10^4^	2.14	70.5	61.8	108.4	62.7
PBSAD-14	14 mol%	2.48 × 10^4^	1.84	61.1	49.8	101.1	50.5

**Table 2:** Isothermal crystallization kinetics parameters of PBS and PBSAD.

Samples	*T*_c_ (°C)	*n*	*k* (s^−*n*^)	*t*_1/2_ (s)
PBS	93	1.84	9.33 × 10^−5^	127
	95	1.83	5.89 × 10^−^^5^	168
	97	1.76	3.89 × 10^−^^5^	260
	99	1.74	1.35 × 10^−^^5^	510
PBSAD-5	87	1.91	2.24 × 10^−5^	225
	89	1.92	1.32 × 10^−5^	288
	91	2.15	7.76 × 10^−7^	586
	93	2.45	2.95 × 10^−8^	1020
PBSAD-7	85	1.87	4.90 × 10^−^^5^	166
	87	1.81	2.51 × 10^−^^5^	284
	89	1.97	2.95 × 10^−^^6^	533
	91	2.15	3.02 × 10^−^^7^	909
PBSAD-14	76	1.91	5.37 × 10^−^^5^	142
	78	1.83	3.31 × 10^−^^5^	230
	80	1.84	1.29 × 10^−^^5^	373
	82	1.98	2.24 × 10^−^^6^	593

**Table 3:** Nucleation densities (*N*) and average spherulite diameters (*d*) of PBS and PBSAD.

Sample	*N* (pcs/mm^2^)	*d* (μm)
PBS	~56	~151
PBSAD-5	~159	~89
PBSAD-7	~1101	~34
PBSAD-14	~2963.0	~21

**Table 4:** The temperature ranges of domains *II*, *II*_a_, and *II*_b_ of PBS and PBSAD.

Sample	Domain *II* (°C)	Domain *II*_a_ (°C)	Domain *II*_b_ (°C)
PBS	15.0	10.5	4.5
PBSAD-5	11.0	6.5	4.5
PBSAD-7	9.0	5.0	4.0
PBSAD-14	6.5	2.3	4.2

## Data Availability

The data presented in this study are available on request from the corresponding author.

## Supplementary Materials

The following are available online at https://www.mdpi.com/2073-4360/13/3/365/s1. Figure S1: The ^1^H NMR spectra of PBSAD-5 and PBSAD-7. Figure S2: The relative crystallinity development trend of PBS and PBSAD. Figure S3: Avrami plots for PBS and PBSAD. Figure S4: The spherulites morphology of PBSFs. Figure S5: The cooling DSC curves of polyesters after being in melted at various *T*_s_ for 5 min.

## References

[1] Xu J., Guo B.H. (2010). Poly(butylene succinate) and its copolymers: Research, development and industrialization. Biotechnol. J..

[2] Ishioka R., Kitakuni E., Ichikawa Y., Doi Y., Steinbüchel A. (2002). Aliphatic polyesters: “Bionolle”. Biopolymers.

[3] Sisti L., Totaro G., Marchese P., Kalia S., Averous L. (2016). PBS makes its entrance into the family of biobased plastics. Biodegradable and Biobased Polymers for Environmental and Biomedical Applications.

[4] Xu J., Guo B.H., Chen G.Q. (2016). Microbial Succinic Acid, Its Polymer Poly(butylene succinate), and Applications. Plastics from Bacteria: Natural Functions and Applications.

[5] Fujimaki T. (1998). Processability and properties of aliphatic polyesters, ‘BIONOLLE’, synthesized by polycondensation reaction. Polym. Degrad. Stabl..

[6] Ye H.M., Tang Y.R., Xu J., Guo B.H. (2013). Role of Poly(butylene fumarate) on Crystallization Behavior of Poly(butylene succinate). Ind. Eng. Chem. Res..

[7] Yang B., Ni H., Huang J., Luo Y. (2014). Effects of Poly(vinyl butyral) as a Macromolecular Nucleating Agent on the Nonisothermal Crystallization and Mechanical Properties of Biodegradable Poly(butylene succinate). Macromolecules.

[8] Tang Y.R., Lin D.W., Gao Y., Xu J., Guo B.H. (2014). Prominent Nucleating Effect of Finely Dispersed Hydroxyl-Functional Hexagonal Boron Nitride on Biodegradable Poly(butylene succinate). Ind. Eng. Chem. Res..

[9] Wei Z., Chen G., Shi Y. (2012). Isothermal crystallization and mechanical properties of poly(butylene succinate)/layered double hydroxide nanocomposites. J. Polym. Res..

[10] Yarici T., Kodal M., Ozkoc G. (2018). Non-isothermal crystallization kinetics of Poly(Butylene succinate) (PBS) nanocomposites with different modified carbon nanotubes. Polymer.

[11] Bosq N., Aht-Ong D. (2018). Isothermal and non-isothermal crystallization kinetics of poly(butylene succinate) with nanoprecipitated calcium carbonate as nucleating agent. J. Therm. Anal. Calorim..

[12] Kim H.S., Yang H.S., Kim H.J. (2005). Biodegradability and mechanical properties of agroflour–filled polybutylene succinate biocomposites. J. Appl. Polym. Sci..

[13] Li J., Luo X., Lin X. (2013). Preparation and characterization of hollow glass microsphere reinforced poly(butylene succinate) composites. Mater. Des..

[14] Nikolic M.S., Djonlagic J. (2001). Synthesis and characterization of biodegradable poly(butylene succinate-co-butylene adipate)s. Polym. Degrad. Stabl..

[15] Li F., Xu X., Hao Q., Li Q., Yu J., Cao A. (2006). Effects of comonomer sequential structure on thermal and crystallization behaviors of biodegradable poly(butylene succinate-co-butylene terephthalate)s. J. Polym. Sci. Part B Polym. Phys..

[16] Xu Y., Xu J., Guo B., Xie X. (2007). Crystallization kinetics and morphology of biodegradable poly(butylene succinatecopropylene succinate)s. J. Polym. Sci. B Polym. Phys..

[17] Mochizuki M., Mukai K., Yamada K., Ichise N., Murase S., Iwaya Y. (1997). Structural Effects upon Enzymatic Hydrolysis of Poly(butylene succinate-co-ethylene succinate)s. Macromolecules.

[18] Liu X.Q., Li C.C., Zhang D., Xiao Y.N. (2006). Melting behaviors, crystallization kinetics, and spherulitic morphologies of poly(butylene succinate) and its copolyester modified with rosin maleopimaric acid anhydride. J. Polym. Sci. B Polym. Phys..

[19] Yang Y., Qiu Z. (2011). Crystallization kinetics and morphology of biodegradable poly(butylene succinate-co-ethylene succinate) copolyesters: Effects of comonomer composition and crystallization temperature. CrystEngComm.

[20] Sun Z., Jiang Z., Qiu Z. (2020). Thermal, crystallization and mechanical properties of branched Poly(butylene succinate) copolymers with 1,2-decanediol being the comonomer. Polymer.

[21] Wang L., Zhang M., Lawson T., Kanwal A., Miao Z. (2019). Poly(butylene succinate-co-salicylic acid) copolymers and their effect on promoting plant growth. Roy. Soc. Open Sci..

[22] Zhou X.M., Wang X.Y., Wu T. (2020). Preparation, crystallization and degradation properties of poly(butylene succinate-co-neopentyl glycol succinate) copolymer/graphite oxide composites. J. Therm. Anal. Calorim..

[23] Qi Z., Ye H., Xu J., Chen J., Guo B. (2013). Improved the thermal and mechanical properties of poly(butylene succinate-co-butylene adipate) by forming nanocomposites with attapulgite. Colloids Surf. A.

[24] Ye H.M., Wang R.D., Liu J., Xu J., Guo B.H. (2012). Isomorphism in Poly(butylene succinate-co-butylene fumarate) and Its Application as Polymeric Nucleating Agent for Poly(butylene succinate). Macromolecules.

[25] Zeng J.B., Wu F., Huang C.L., He Y.S., Wang Y.Z. (2012). Urethane Ionic Groups Induced Rapid Crystallization of Biodegradable Poly(ethylene succinate). ACS Macro Lett..

[26] Tabata H., Fujii M., Hayashi S., Doi T., Wakabayashi T. (2006). Raman and surface-enhanced Raman scattering of a series of size-separated polyynes. Carbon.

[27] Li S., Chen T., Wang Y., Liu L., Lv F., Li Z., Huang Y., Schanze K.S., Wang S. (2017). Conjugated Polymer with Intrinsic Alkyne Units for Synergistically Enhanced Raman Imaging in Living Cells. Angew. Chem. Int. Ed..

[28] Wei L., Hu F., Shen Y., Chen Z., Yu Y., Lin C.C., Wang M.C., Min W. (2014). Live-cell imaging of alkyne-tagged small biomolecules by stimulated Raman scattering. Nat. Methods.

[29] Mustapha A., Salga M.S., Sabo S. (2018). Synthesis and characterisation of some mixed ligands adducts of benzoylacetone and salicylaldehyde. J. Pure Appl. Sci..

[30] Abood Z.H., Haiwal R.T., Kadum I.L., Gzar K.O., Radhi S.M., Hameem R.K., Abbas S.K. (2008). Synthesis of Some New Azo Schiff Bases and Tetrazole Derivatives from 2-Amino-1,3,4-thiadiazole-5-thiol. J. Kerbala Univ..

[31] Tserki V., Matzinos P., Pavlidou E., Vachliotis D., Panayiotou C. (2006). Biodegradable aliphatic polyesters. I. Properties and biodegradation of poly(butylene succinate-co-butylene adipate). Polym. Degrad. Stabl..

[32] Papageorgiou G.Z., Bikiaris D.N. (2007). Synthesis, Cocrystallization, and Enzymatic Degradation of Novel Poly(butylene-co-propylene succinate) Copolymers. Biomacromolecules.

[33] Nagata M., Goto H., Sakai W., Tsutsumi N. (2000). Synthesis and enzymatic degradation of poly(tetramethylene succinate), copolymers with terephthalic acid. Polymer.

[34] Avrami M. (1939). Kinetics of Phase Change. I. General Theory. J. Chem. Phys..

[35] Avrami M. (1941). Granulation, Phase Change, and Microstructure Kinetics of Phase Change. III. J. Chem. Phys..

[36] Dai X., Qiu Z. (2017). Crystallization kinetics, morphology, and hydrolytic degradation of novel biobased poly(butylene succinate-co-decamethylene succinate) copolyesters. Polym. Degrad. Stabl..

[37] Mao H.I., Chen C.W., Rwei S.P. (2020). Synthesis and Nonisothermal Crystallization Kinetics of Poly(Butylene Terephthalate-co-Tetramethylene Ether Glycol) Copolyesters. Polymers.

[38] Lu X., Wen X., Yang D. (2011). Isothermal crystallization kinetics and morphology of biodegradable poly(3-hydroxybutyrate-co-4-hydroxybutyrate). J. Mater. Sci..

[39] Wang T., Wang H., Li H., Gan Z., Yan S. (2009). Banded spherulitic structures of poly(ethylene adipate), poly(butylene succinate) and in their blends. Phys. Chem. Chem. Phys..

[40] Yoo E.S., Im S.S. (1999). Melting behavior of poly(butylene succinate) during heating scan by DSC. J. Polym. Sci. B Polym. Phys..

[41] Jiang J., Zhuravlev E., Hu W.B., Schick C., Zhou D.S. (2017). The effect of self-nucleation on isothermal crystallization kinetics of poly(butylene succinate) (PBS) investigated by differential fast scanning calorimetry. Chinese J. Polym. Sci..

[42] Sangroniz L., Cavallo D., Müller A.J. (2020). Self-Nucleation Effects on Polymer Crystallization. Macromolecules.

[43] Ye H.M., Liu P., Wang C.X., Meng X., Zhou Q. (2017). Polymorphism regulation in Poly(hexamethylene succinate-co-hexamethylene fumarate): Altering the hydrogen bonds in crystalline lattice. Polymer.

[44] Meng X.Y., Li Y., Yao S.F., Wei X.W., Ye H.M. (2020). Unusual Spherulitic Morphology of Poly(propylene fumarate). Chinese J. Polym. Sci..

[45] Ye H.M., Wang J., Wang C.S., Li H.F. (2019). Unique Isodimorphism of Poly(decamethylene succinate-ran-decamethylene fumarate): Large Pseudoeutectic Region and Fantastic Crystallization/Melting Behavior. Macromolecules.

[46] Tang X., Chen W., Li L. (2019). The Tough Journey of Polymer Crystallization: Battling with Chain Flexibility and Connectivity. Macromolecules.

