# Supplementary material for: Poly(butylene succinate-co-butylene acetylenedicarboxylate): Copolyester with Novel Nucleation Behavior
Source: Polymers (Basel). 2021 Jan 24;13(3):365. doi: 10.3390/polym13030365 (PMC7865284; doi:10.3390/polym13030365)
Supplement: Supplementary file 1 [file polymers-13-00365-s001.pdf]

Poly(butylene succinate-*co*-butylene acetylenedicarboxylate): Copolyester with Novel Nucleation Behavior

Yi Li, Guoyong Huang, Cong Chen, Xue-Wei Wei, Xi Dong, Wei Zhao, Hai-Mu Ye

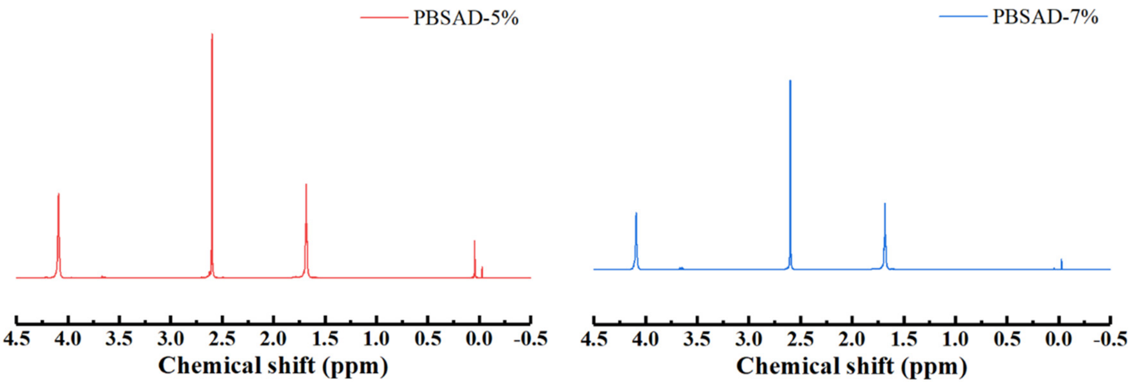

Figure S1. The ^{1}H NMR spectra of PBSAD-5 and PBSAD-7. The spectrum of PBSAD-14 is presented in main text.

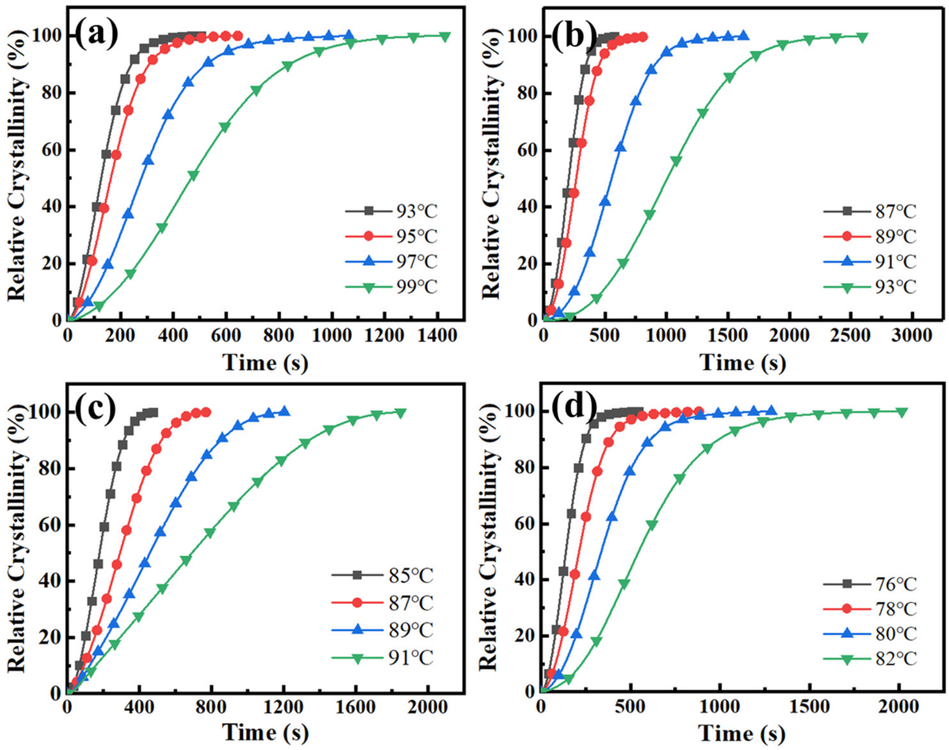

Figure S2. The relative crystallinity development trend of (a) PBS, (b) PBSAD-5, (c) PBSAD-7 and (d) PBSAD-14.

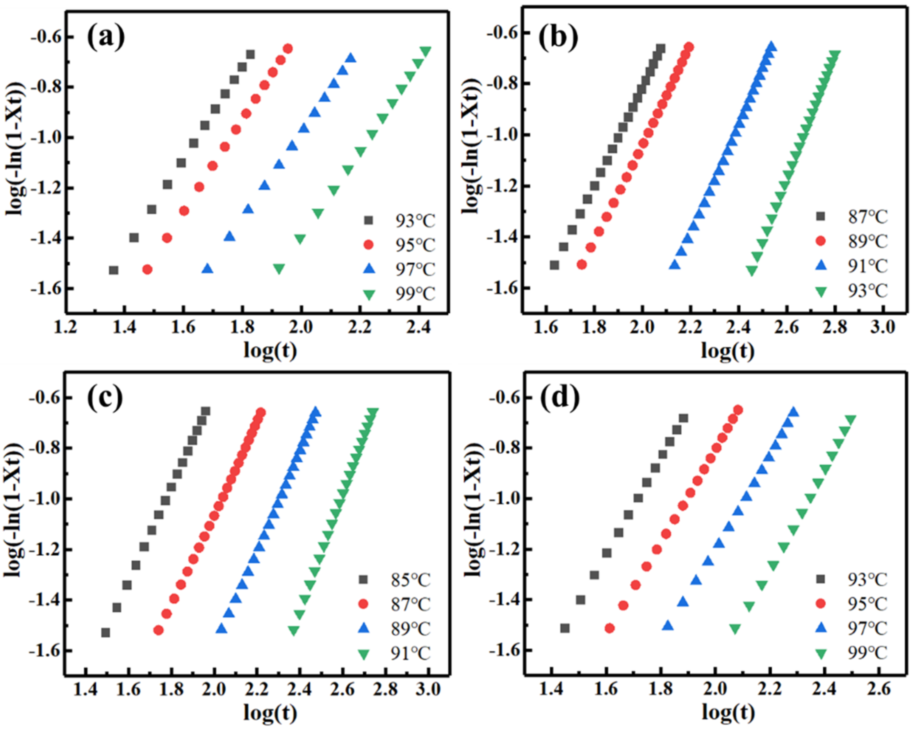

Figure S3. Avrami plots for (a) PBS, (b) PBSAD-5, (c) PBSAD-7 and (d) PBSAD-14.

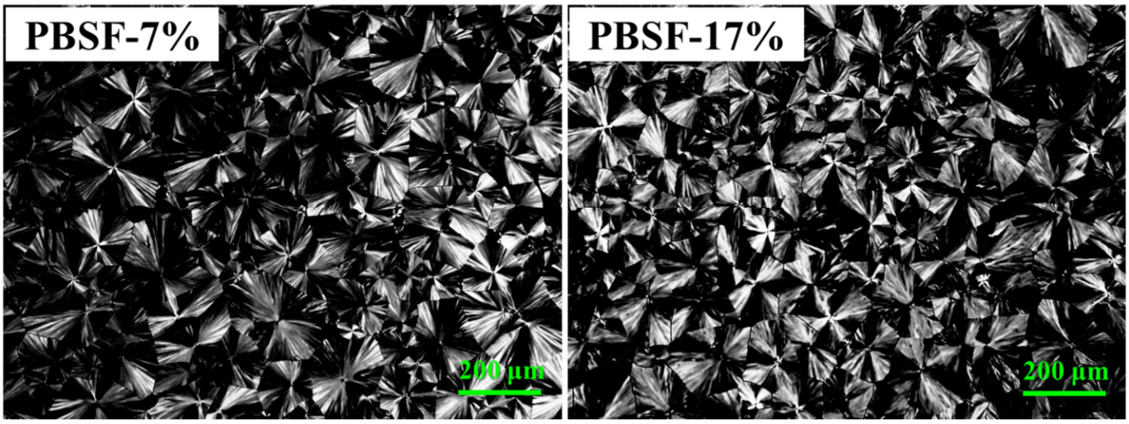

Figure S4. The spherulites morphology of PBSFs, which have similar molecular weight is PBSAD.

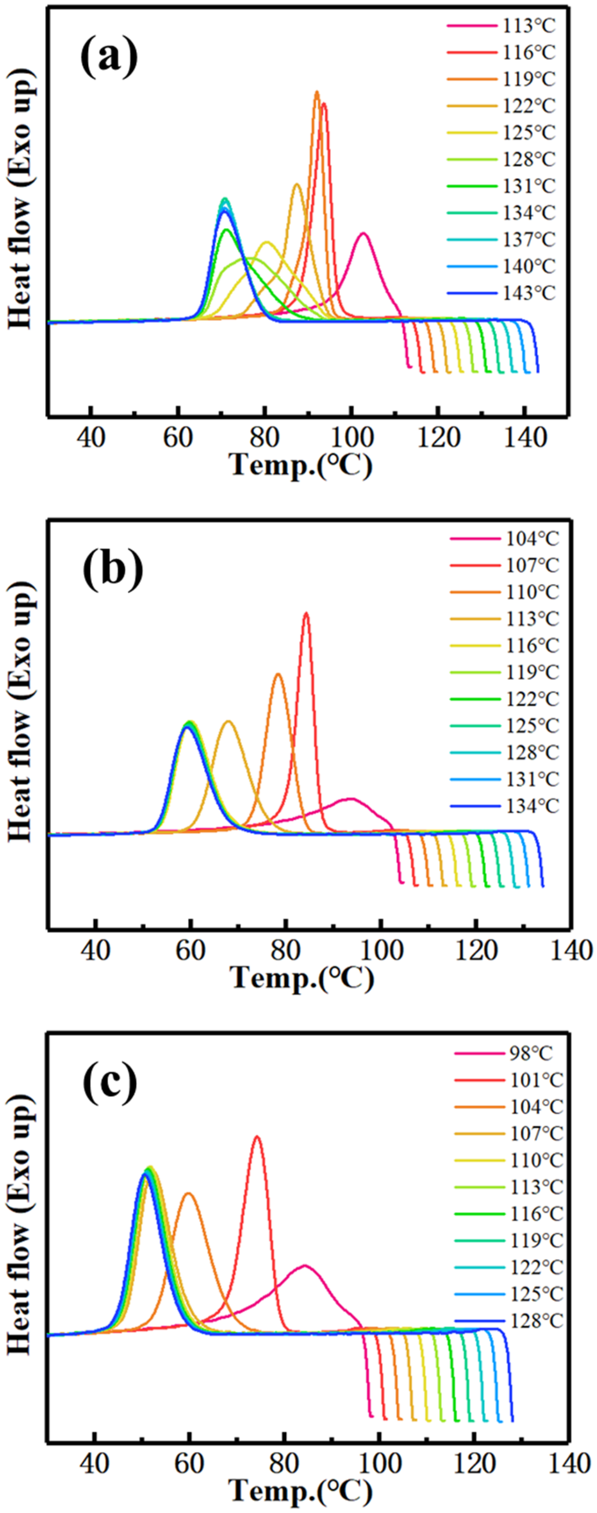

Figure S5. The cooling DSC curves of polyesters after being in melted at various T_s for 5 min. (a) PBS (b) PBSAD-7 and (c) PBSAD-14.
